# Maternal Characteristics and Prevalence of Infants Born Small for Gestational Age

**DOI:** 10.1001/jamanetworkopen.2024.29434

**Published:** 2024-08-21

**Authors:** Liangcheng Xiang, Xiaohong Li, Yi Mu, Peiran Chen, Yanxia Xie, Yanping Wang, Li Dai, Zheng Liu, Qi Li, Mingrong Li, Juan Liang, Jun Zhu

**Affiliations:** 1 National Office for Maternal and Child Health Surveillance of China, West China Second University Hospital, Sichuan University, Chengdu, Sichuan, China; 2 Key Laboratory of Birth Defects and Related Diseases of Women and Children, Sichuan University, Ministry of Education, Chengdu, Sichuan, China; 3 Sichuan Birth Defects Clinical Research Center, West China Second University Hospital, Sichuan University, Chengdu, Sichuan, China; 4 Department of Pediatrics, West China Second University Hospital, Sichuan University, Chengdu, Sichuan, China; 5 Department of Obstetrics, West China Second University Hospital, Sichuan University, Chengdu, Sichuan, China

## Abstract

**Importance:**

Being born small for gestational age (SGA) is a risk factor for neonatal mortality and adverse outcomes in the short and long term. The maternal profile in China has substantially changed over the past decade, which may affect the risk of infants born SGA.

**Objectives:**

To analyze the prevalence of infants born SGA from 2012 through 2020 and explore the association of maternal sociodemographic characteristics and other factors with that prevalence.

**Design, Setting, and Participants:**

This cross-sectional study examined data from the National Maternal Near Miss Surveillance System on women who delivered singleton live births at gestational ages of 28 to 42 weeks from January 1, 2012, through December 31, 2020, in China. Statistical analysis was performed from December 2022 to September 2023.

**Exposures:**

Characteristics of delivery (year, region of country, and hospital level), mother (age, educational level, marital status, prenatal visits, parity, preexisting diseases, or prenatal complications), and newborn (birth weight, sex, and gestational age).

**Main Outcomes and Measures:**

Prevalence of infants born SGA stratified by severity and by region of the country, changes in prevalence based on log-linear Poisson regression with robust variance, and association of maternal characteristics with changes in prevalence of infants born SGA between 2012 and 2020 based on the Fairlie nonlinear mean decomposition.

**Results:**

Among 12 643 962 births (6 572 548 [52.0%] male; median gestational age, 39 weeks [IQR, 38-40 weeks]), the overall weighted prevalence of infants born SGA was 6.4%, which decreased from 7.3% in 2012 to 5.3% in 2020, translating to a mean annual decrease rate of 3.9% (95% CI, 3.3%-4.5%). The prevalence of infants born SGA decreased from 2.0% to 1.2% for infants with severe SGA birth weight and from 5.3% to 4.1% for those with mild to moderate SGA birth weight. The mean annual rate of decrease was faster for infants with severe SGA birth weight than for those with mild to moderate SGA birth weight (5.9% [95% CI, 4.6%-7.1%] vs 3.2% [95% CI, 2.6%-3.8%]) and was faster for the less developed western (5.3% [95% CI, 4.4%-6.1%]) and central (3.9% [95% CI, 2.9%-4.8%]) regions compared with the eastern region (2.3% [95% CI, 1.1%-3.4%]). Two-thirds of the observed decrease in the prevalence of infants born SGA could be accounted for by changes in maternal characteristics, such as educational level (relative association, 19.7%), age (relative association, 18.8%), prenatal visits (relative association, 20.4%), and parity (relative association, 19.4%). Conversely, maternal preexisting diseases or prenatal complications counteracted the decrease in the prevalence of infants born SGA (−6.7%).

**Conclusions and Relevance:**

In this cross-sectional study of births in China from 2012 to 2020, maternal characteristics changed and the prevalence of infants born SGA decreased. Future interventions to reduce the risk of infants born SGA should focus on primary prevention.

## Key Points

**Question:**

Are maternal characteristics associated with the prevalence of infants born small for gestational age (SGA) in China?

**Findings:**

This cross-sectional study of more than 12.5 million singleton live births at 28 to 42 gestational weeks found that the prevalence of SGA infants decreased from 7.3% in 2012 to 5.3% in 2020. Maternal characteristics associated with this decrease included maternal educational level, age, parity, and prenatal visits, particularly among mothers of mild to moderate SGA newborns and particularly in eastern parts of the country.

**Meaning:**

This study suggests that changes in maternal characteristics were associated with the decrease in the prevalence of SGA infants in China and that the strength of the association varied with geographic region and SGA severity.

## Teaser

This cross-sectional study of more than 12.5 million singleton live births analyzes the prevalence of infants born small for gestational age in China from 2012 through 2020 and examines the association of maternal sociodemographic characteristics and other factors with that prevalence.

## Introduction

Being born small for gestational age (SGA) increases the risk of stillbirth and neonatal mortality, especially in countries with less health infrastructure,^1,2^ and it increases risk of morbidity in the short and long term.^3,4,5,6,7^ Just over 17% of all live births globally involve SGA birth weight,^2^ so reducing the prevalence of SGA birth weight is important in its own right^8^ and for achieving the internationally agreed-on goal of reducing neonatal mortality.^9^

Reducing the future prevalence of infants born SGA requires understanding how the prevalence has evolved until now. Such understanding is lacking for most countries because of insufficient data, including for China, which is home to 20% of the world’s population. The prevalence of infants born SGA across China has been estimated at 4.6%, but this estimate has come from modeling based on data biased toward more economically developed cities.^10,11,12^ Health care infrastructure and access vary strongly across China, making regional assessments critical.

Reducing the prevalence of infants born SGA also requires identifying what factors are associated with its evolution. Many variables have been associated with infants born SGA, including economic conditions, health care quality, access to health care, maternal sociodemographic characteristics, and preexisting diseases or prenatal complications.^13,14,15,16,17,18^ Many of these factors have changed substantially through China’s rapid socioeconomic development and adjustments in the traditional 1-child policy. On one hand, the proportions of mothers aged older than 35 years and who have other pregnancy-related complications have increased,^19,20^ which may be associated with the risk of infants born SGA.^13^ On the other hand, the proportions of mothers with higher educational levels and better access to high-quality health care have also increased, which may decrease the risk of infants born SGA. These considerations argue for a comprehensive assessment of factors associated with infants born SGA in China.

To address these knowledge gaps, we drew on a comprehensive national database in China to analyze singleton live births across the country from 2012 through 2020. Our aim was to evaluate the prevalence of infants born SGA for the entire country but also by region and by SGA severity, whether mild to moderate or severe. We wanted to assess how the prevalence of infants born SGA has changed during the study period and identify which maternal characteristics may have been associated with those changes. The results of our analysis may guide future public health interventions and health care policy.

## Methods

### Data Source

This cross-sectional study was approved by the Ethics Committee of West China Second University Hospital in Chengdu, China, which waived the requirement for informed consent because the data had been collected with mothers’ consent through government-approved procedures and were maintained in a government-curated database. This study was reported according to the Strengthening the Reporting of Observational Studies in Epidemiology (STROBE) reporting guideline.

Data came from the National Maternal Near Miss Surveillance System (NMNMSS) database in China,^19,20,21,22^ from January 1, 2012, through December 31, 2020. The NMNMSS covered 326 urban districts and rural counties in 30 provinces in mainland China (excluding Tibet). At each surveillance site, 2 health facilities reporting more than 1000 births per year were randomly selected for inclusion, or 1 facility if only 1 was available (eAppendix 1 in Supplement 1). The NMNMSS contains 438 health facilities at the county level or above. Given that nearly all births in China occur in hospitals, the NMNMSS can be estimated to cover approximately 10% of all births in China every year.

The NMNMSS is biased toward births in urban areas because hospitals in some rural areas did not report a sufficient number of annual births to be included in the database. To correct for this urban sampling bias, we weighted data according to the distribution of live births between urban and rural settings in the given year. The distributions in 2010 and 2020 were taken from national census data, and the distributions in 2012 to 2019 were estimated through linear interpolation (eAppendix 2 and eTable 1 in Supplement 1).

Data in the NMNMSS were prospectively collected from mothers before or after birth by obstetric departments in participating hospitals before hospital discharge. Specially trained physicians attending the women completed a specially designed data collection form. Data from each hospital were entered into a web-based reporting system centralized at the National Office for Maternal and Child Health Surveillance (Chengdu, China); data collection and quality control have been detailed elsewhere.^20,21^

### Definitions of Variables

Maternal educational level was categorized as up to primary school, middle school, high school, or college or higher. Maternal marital status was categorized as married or single, widowed, or divorced. Maternal age at delivery was categorized as younger than 20, 20 to 24, 25 to 29, 30 to 34, or older than 34 years. Prenatal visits were categorized as fewer than 5, 5 to 9, 10 to 14, or more than 14. Parity before the current pregnancy was categorized as primiparous or multiparous and did not differentiate between live births or stillbirths. Mothers were assigned to 1 of 3 categories of complications: (1) prenatal complications, including placenta previa, placental abruption, chronic hypertension, gestational hypertension, preeclampsia, eclampsia, or HELLP (hemolysis, elevated liver enzymes, and low platelets) syndrome; (2) preexisting disease, including heart disease, embolism, hepatic disease, severe anemia (hemoglobin concentration <7.0 g/dL [to convert to grams per liter, multiply by 10.0]), diabetes, urinary tract infection or other kidney disease, upper respiratory tract infection or other lung disease, HIV, connective tissue disorder, cancer, hypothyroidism, syphilis; or (3) none of these. Women who had preexisting disease and who experienced prenatal complications were assigned only to the prenatal complications category.

Some analyses were stratified by eastern, central, or western regions of China based on the regions defined by the China Maternal and Child Health Statistics Standards.^23^ This stratification reflected the substantial geographic disparities in economic development across the country; the median per capita gross domestic product is $8546 in 2020 US dollars (USD) in the eastern region, $4394 USD in the central region, and $3949 USD in the western region.^23^

The quality of the health care infrastructure at the delivering hospitals was categorized in terms of a hospital level from 1 to 3, with 3 indicating the highest quality. Gestational age was estimated from the last menstrual period or, if this was unknown, from prenatal ultrasonography. Births were categorized as preterm if they occurred below the gestational age of 37 weeks; otherwise, they were categorized as term.

Birth weight was measured within 1 hour after birth. Small for gestational age weight for all years was defined as birth weight below the 10th percentile according to the INTERGROWTH-21st standards.^24,25^ The severity of SGA birth weight was categorized as mild to moderate if birth weight fell between the 3rd and 10th percentiles (≥3% and <10%) or severe if birth weight fell below the 3rd percentile (<3%).^1,26,27^

### Statistical Analysis

Statistical analysis was performed from December 2022 to September 2023. Data were missing on educational level for 249 683 mothers (2.0%); on marital status for 2277 mothers (0.02%); on age for 2886 mothers (0.02%); on prenatal visits for 412 898 mothers (3.3%); and on parity for 3780 mothers (0.03%). These variables were analyzed by assigning missing data as a separate level. Data were analyzed using Stata, version 16.0 (StataCorp LLC), and results associated with a 2-tailed *P* < .05 were considered statistically significant.

The prevalence of infants born SGA was calculated by dividing the number of SGA births by the total number of births. The prevalence was then weighted by the distribution of live births between urban and rural settings. The evolution of prevalence during the 9-year study period was modeled using log-linear Poisson regression models with robust variance,^28^ which led to a rate ratio that was subtracted from 1 to yield the mean annual rate of decrease for the study period. Where appropriate, results were reported together with 95% CIs.

We explored potential associations of variables with infants born SGA using logistic regression that accounted for the distribution of live births between urban and rural settings and for clustering of births within hospitals. The resulting odds ratios were adjusted for the following variables in multivariable logistic regression: region, hospital level, maternal educational level, maternal marital status, maternal age, prenatal visits, parity, preexisting diseases or prenatal complications, sex, and births.

The association of maternal characteristics with the observed change in the prevalence of infants born SGA between 2012 and 2020 was estimated using the nonlinear Fairlie extension^29,30^ of the regression-based decomposition approach of Blinder and Oaxaca,^31,32,33^ to be able to account for the dichotomy of the dependent variable, which was SGA birth weight.^34^ The independent variables defined in the previous subsection were ordered randomly in the nonlinear decomposition model, and modeling was performed 1000 times to minimize the association of path dependence. Decomposition modeling was performed using the coefficients estimated by the full sample over the entire 9-year study period to minimize the association of sample weighting. The robustness of these analyses was checked by repeating them after defining mild to moderate or severe SGA birth weight according to growth standards from China’s National Health Commission.^35^

## Results

We restricted our analysis to pregnancies with singleton live births from January 1, 2012, through December 31, 2020. Of the 12 735 055 singleton live births in the NMNMSS during the study period, we excluded 91 093 for various reasons (eFigure in Supplement 1), leaving 12 643 962 newborns delivered at gestational ages of 28 to 42 weeks and their mothers in the final analysis. Of the newborns, 6 572 548 (52.0%) were male, and their median gestational age was 39 weeks (IQR, 38-40 weeks).

### Temporal Changes in SGA Birth Weight

A total of 791 986 newborns were SGA, corresponding to a weighted prevalence of 6.4% (Table 1). Of these SGA births, 202 731 (25.6%) were severe, corresponding to a weighted prevalence of 1.6%; and 589 255 (74.4%) were mild or moderate, corresponding to a weighted prevalence of 4.8%. The weighted prevalence of infants born SGA decreased from 7.3% in 2012 to 5.3% in 2020 for the 2 categories of severity combined, from 2.0% to 1.2% for infants with severe SGA birth weight and from 5.3% to 4.1% for those with mild to moderate SGA birth weight. The mean annual rates of decrease were 3.9% (95% CI, 3.3%-4.5%) for both categories of SGA severity combined, 5.9% (95% CI, 4.6%-7.1%) for infants with severe SGA birth weight and 3.2% (95% CI, 2.6%-3.8%) for those with mild to moderate SGA birth weight.

**Table 1.** Prevalence of Infants Born SGA in China, 2012-2020

Geographic area and SGA severity	Infants born SGA, No. (weighted % of total)^a^										Annual decrease rate, mean (95% CI), %^b^
	Entire period (N = 12 643 962)	2012 (n = 1 262 022)	2013 (n = 1 230 539)	2014 (n = 1 407 846)	2015 (n = 1 245 202)	2016 (n = 1 471 440)	2017 (n = 1 571 371)	2018 (n = 1 335 381)	2019 (n = 1 993 590)	2020 (n = 1 126 571)	
**Entire country**											
All severities	791 986 (6.4)	89 766 (7.3)	88 570 (7.3)	96 346 (6.9)	85 801 (6.9)	89 569 (6.1)	94 749 (6.1)	77 349 (6.0)	111 110 (5.7)	58 726 (5.3)	3.9 (3.3-4.5)
Severe	202 731 (1.6)	24 744 (2.0)	24 100 (2.0)	25 491 (1.8)	22 281 (1.8)	22 577 (1.5)	23 296 (1.5)	19 048 (1.4)	27 415 (1.4)	13 779 (1.2)	5.9 (4.6-7.1)
Mild to moderate	589 255 (4.8)	65 022 (5.3)	64 470 (5.3)	70 855 (5.1)	63 520 (5.1)	66 992 (4.6)	71 453 (4.6)	58 301 (4.6)	83 695 (4.3)	44 947 (4.1)	3.2 (2.6-3.8)
**Eastern region**											
All severities	183 935 (5.5)	20 383 (5.8)	19 668 (6.0)	22 989 (5.7)	20 008 (5.9)	20 396 (5.3)	22 053 (5.4)	18 494 (5.3)	26 249 (5.2)	13 695 (4.8)	2.3 (1.1-3.4)
Severe	42 594 (1.3)	5176 (1.4)	4892 (1.5)	5557 (1.4)	4690 (1.4)	4740 (1.2)	4878 (1.2)	4055 (1.1)	5709 (1.1)	2897 (1.0)	4.3 (2.9-5.7)
Mild to moderate	141 341 (4.2)	15 207 (4.4)	14 776 (4.5)	17 432 (4.3)	15 318 (4.5)	15 656 (4.1)	17 175 (4.2)	14 439 (4.2)	20 540 (4.1)	10 798 (3.8)	1.6 (0.4-2.8)
**Central region**											
All severities	287 624 (5.8)	32 196 (6.6)	31 735 (6.5)	34 709 (6.3)	30 019 (6.2)	31 790 (5.5)	33 519 (5.5)	29 402 (5.5)	42 168 (5.2)	22 086 (4.6)	3.9 (2.9-4.8)
Severe	74 558 (1.5)	8982 (1.8)	8600 (1.7)	9218 (1.6)	7853 (1.6)	7985 (1.3)	8097 (1.3)	7439 (1.4)	10 980 (1.3)	5404 (1.1)	5.0 (1.8-8.2)
Mild to moderate	213 066 (4.3)	23 214 (4.8)	23 135 (4.8)	25 491 (4.7)	22 166 (4.6)	23 805 (4.2)	25 422 (4.2)	21 963 (4.1)	31 188 (3.9)	16 682 (3.5)	3.5 (2.9-4.1)
**Western region**											
All severities	320 427 (8.3)	37 187 (10.0)	37 167 (9.9)	38 648 (9.3)	35 774 (9.1)	37 383 (8.0)	39 177 (7.8)	29 453 (7.5)	42 693 (7.0)	22 945 (6.7)	5.3 (4.4-6.1)
Severe	85 579 (2.2)	10 586 (2.9)	10 608 (2.9)	10 716 (2.6)	9738 (2.5)	9852 (2.1)	10 321 (2.1)	7554 (1.9)	10 726 (1.8)	5478 (1.6)	7.6 (6.4-8.7)
Mild to moderate	234 848 (6.1)	26 601 (7.1)	26 559 (7.0)	27 932 (6.7)	26 036 (6.6)	27 531 (5.9)	28 856 (5.7)	21 899 (5.6)	31 967 (5.2)	17 467 (5.1)	4.4 (3.6-5.2)

Abbreviation: SGA, small for gestational age.

^a^ Adjusted for the distribution of live births between urban and rural settings.

^b^ Mean annual decrease rate over the entire period, as estimated by log-linear Poisson regression involving robust variance and adjustment for distribution of live births between urban and rural settings and for clustering of births within hospitals.

The weighted prevalence of infants born SGA during the study period was highest in the western region (8.3%). The mean annual rate of decrease was also highest in the western region (5.3% [95% CI, 4.4%-6.1%]) and central region (3.9% [95% CI, 2.9%-4.8%]) compared with the eastern region (2.3% [95% CI, 1.1%-3.4%]). In all 3 regions, prevalence decreased faster for infants with severe SGA birth weight than for those with mild to moderate SGA birth weight.

### Maternal Characteristics and Decrease of SGA Birth Weight

**Figure 1.**
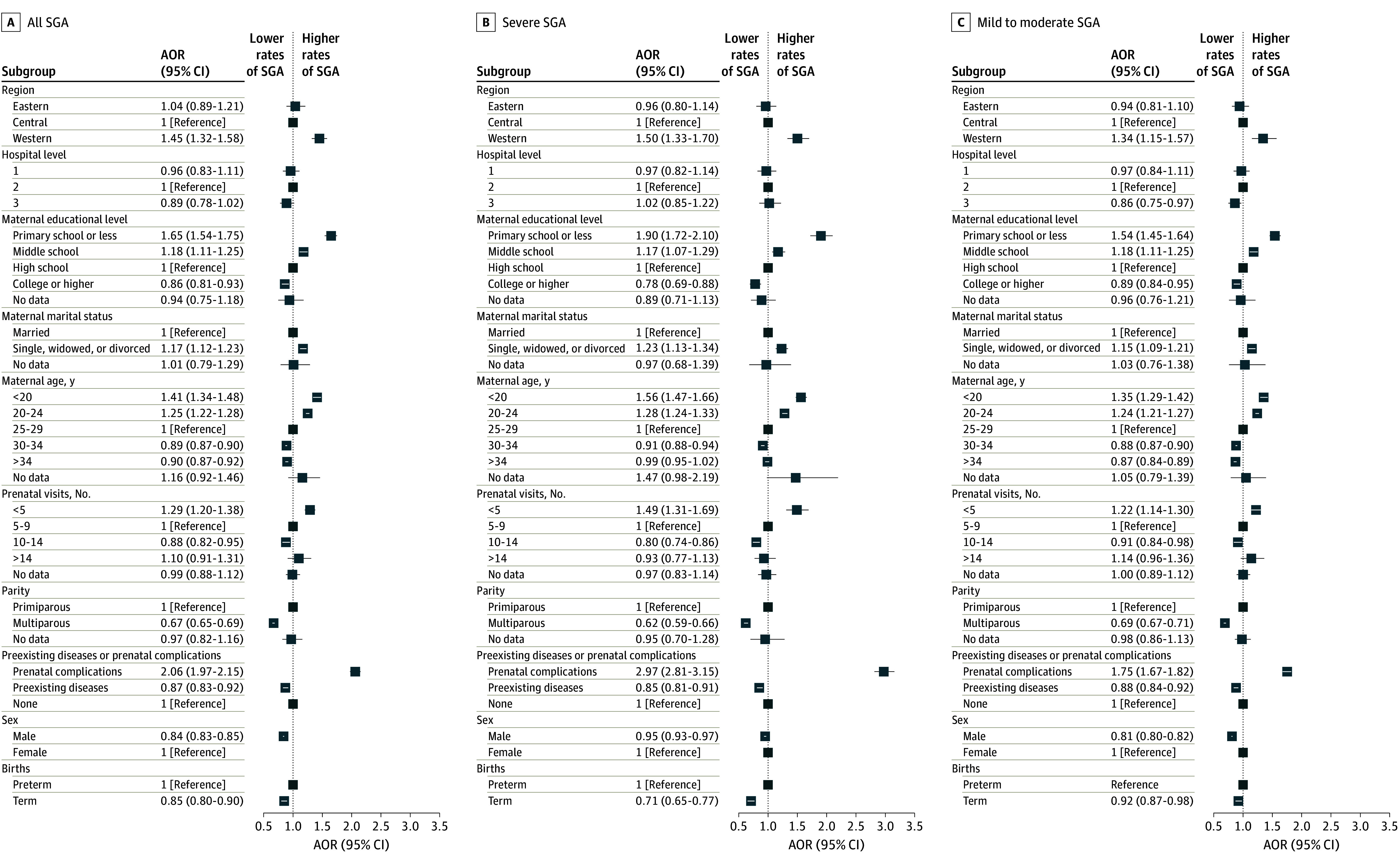
Forest Plots of the Risk of Small for Gestational Age (SGA) Birth Weight Risk was calculated separately for all SGA infants, severe SGA birth weight, or mild to moderate SGA birth weight. All models adjusted for the distribution of live births between urban and rural settings, the clustering of births within hospitals, and region, hospital level, maternal educational level, maternal marital status, maternal age, prenatal visits, parity, preexisting disease or prenatal complications, sex, and births. AOR indicates adjusted odds ratio.

**Figure 2.**
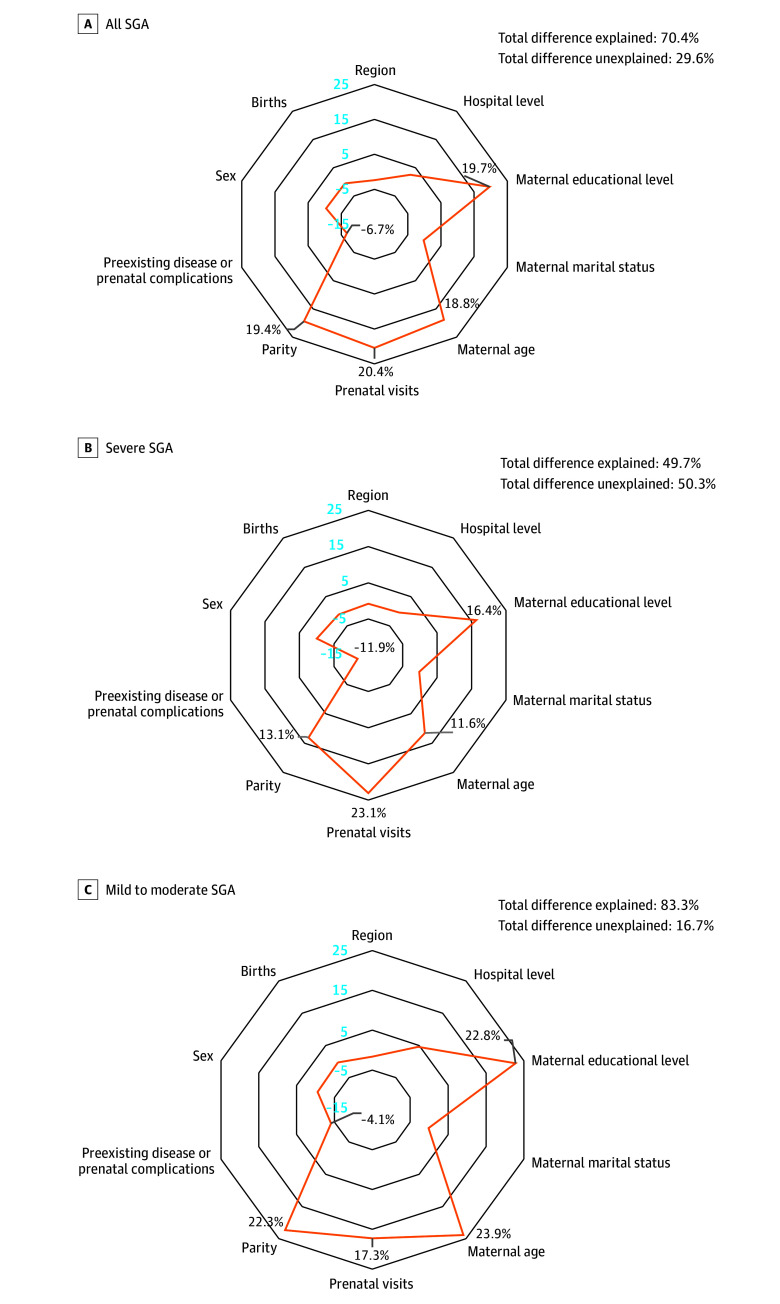
Radar Charts of Relative Associations of Maternal Characteristics With Changes in Prevalence of Small for Gestational Age (SGA) Birth Weight The numbers in blue represent the tick marks of the coordinate axis.

Small for gestational age birth weight was significantly associated with prenatal visits as well as educational level, age, parity, and preexisting disease or prenatal complications in the mother (Figure 1; Table 2). Changes in maternal characteristics accounted for two-thirds of the observed decrease (2.0 per 100 live births) in prevalence between 2012 and 2020; the characteristics most associated with the prevalence of infants born SGA were maternal educational level (relative association, 19.7%), age (relative association, 18.8%), and parity (relative association, 19.4%), as well as prenatal visits (relative association, 20.4%) (Figure 2). The proportions of women with lower educational level, younger age, primiparity, or fewer prenatal visits, all of which are associated with higher risk of infants born SGA, decreased during the study period. In parallel, the proportions of women with higher educational level, older age, multiparity, or more prenatal visits, all of which are associated with lower risk of infants born SGA, increased during the study period. The association of these variables with the decrease in prevalence of infants born SGA was slightly counteracted by the increase in proportions of women with preexisting disease or prenatal complications (relative association, –6.7%).

**Table 2.** Distribution of Maternal and Other Characteristics in China, 2012-2020

Characteristic	No. (%)									Difference, 2012-2020, %
	2012 (n = 1 262 022)	2013 (n = 1 230 539)	2014 (n = 1 407 846)	2015 (n = 1 245 202)	2016 (n = 1 471 440)	2017 (n = 1 571 371)	2018 (n = 1 335 381)	2019 (n = 1 993 590)	2020 (n = 1 126 571)	
Region										
Eastern	373 085 (29.6)	351 388 (28.6)	421 058 (29.9)	360 508 (29.0)	425 127 (28.9)	447 257 (28.5)	377 768 (28.3)	561 184 (28.2)	309 522 (27.5)	−2.1
Central	500 078 (39.6)	491 125 (39.9)	560 791 (39.8)	488 389 (39.2)	586 470 (39.9)	615 761 (39.2)	541 209 (40.5)	795 372 (39.9)	455 564 (40.4)	0.8
Western	388 859 (30.8)	388 026 (31.5)	425 997 (30.3)	396 305 (31.8)	459 843 (31.2)	508 353 (32.3)	416 404 (31.2)	637 034 (31.9)	361 485 (32.1)	1.3
Hospital level										
1	159 042 (12.6)	151 879 (12.3)	160 319 (11.4)	144 570 (11.6)	168 302 (11.4)	162 349 (10.3)	130 901 (9.8)	193 043 (9.7)	113 201 (10.0)	−2.6
2	615 171 (48.7)	595 401 (48.4)	671 842 (47.7)	593 808 (47.7)	677 158 (46.0)	703 752 (44.8)	565 709 (42.4)	802 099 (40.2)	456 773 (40.6)	−8.1
3	487 809 (38.7)	483 259 (39.3)	575 685 (40.9)	506 824 (40.7)	625 980 (42.6)	705 270 (44.9)	638 771 (47.8)	998 448 (50.1)	556 597 (49.4)	10.7
Maternal educational level										
Primary school or less	61 463 (4.9)	52 870 (4.3)	52 989 (3.8)	45 947 (3.7)	41 165 (2.8)	41 472 (2.6)	31 631 (2.4)	43 541 (2.2)	26 473 (2.4)	−2.5
Middle school	489 273 (38.8)	460 364 (37.4)	467 059 (33.2)	424 738 (34.1)	433 379 (29.4)	423 252 (26.9)	320 418 (24.0)	426 308 (21.4)	245 836 (21.8)	−17.0
High school	322 557 (25.6)	319 372 (25.9)	384 065 (27.3)	344 708 (27.7)	397 178 (27.0)	432 876 (27.6)	358 839 (26.9)	512 394 (25.7)	283 909 (25.2)	−0.4
College or higher	363 398 (28.8)	368 915 (30.0)	468 198 (33.3)	401 501 (32.2)	574 886 (39.1)	650 648 (41.4)	600 828 (45.0)	973 406 (48.8)	548 423 (48.7)	19.9
No data	25 331 (1.9)	29 018 (2.4)	35 535 (2.4)	28 308 (2.3)	24 832 (1.7)	23 123 (1.5)	23 665 (1.7)	37 941 (1.9)	21 930 (1.9)	0
Maternal marital status										
Married	1 256 292 (99.6)	1 224 506 (99.5)	1 401 201 (99.5)	1 239 478 (99.6)	1 464 323 (99.5)	1 563 164 (99.5)	1 327 550 (99.4)	1 981 331 (99.4)	1 117 902 (99.2)	−0.4
Single, widowed, or divorced	5420 (0.4)	5773 (0.5)	6351 (0.5)	5492 (0.4)	6852 (0.5)	8042 (0.5)	7598 (0.6)	11 850 (0.6)	8560 (0.8)	0.4
No data	310 (0.02)	260 (0.02)	294 (0.02)	232 (0.02)	265 (0.02)	165 (0.01)	233 (0.02)	409 (0.02)	109 (0.01)	−0.1
Maternal age, y										
<20	83578 (6.6)	69334 (5.6)	82626 (5.9)	73596 (5.9)	56955 (3.9)	50911 (3.2)	33 236 (2.5)	44 587 (2.2)	57 108 (5.1)	−1.5
20-24	297 077 (23.6)	277 361 (22.6)	262 972 (18.7)	220 005 (17.7)	211 388 (14.4)	204 353 (13.0)	165 488 (12.4)	221 275 (11.1)	118 391 (10.5)	−13.1
25-29	504 037 (40.0)	499 535 (40.6)	613 681 (43.6)	522 516 (42.0)	639 472 (43.5)	610 386 (38.9)	515 425 (38.6)	749 075 (37.6)	386 815 (34.3)	−5.7
30-34	270 389 (21.4)	269 327 (21.9)	315 729 (22.4)	288 535 (23.2)	380 492 (25.8)	446 685 (28.4)	410 364 (30.7)	680 029 (34.1)	416 689 (37.0)	15.6
>34	106 313 (8.4)	114 527 (9.3)	132 260 (9.4)	140 002 (11.2)	182 637 (12.4)	258 990 (16.5)	210 824 (15.8)	298 564 (15.0)	147 537 (13.1)	4.7
No data	628 (0.05)	455 (0.04)	578 (0.04)	548 (0.04)	496 (0.03)	46 (0.003)	44 (0.003)	60 (0.003)	31 (0.003)	−0.05
Prenatal visits, No.										
<5	259 192 (20.6)	226 702 (18.4)	221 154 (15.7)	178 775 (14.4)	173 516 (11.8)	146 226 (9.3)	101 933 (7.6)	129 833 (6.5)	64 857 (5.8)	−14.8
5-9	684 287 (54.2)	675 118 (54.9)	759 139 (53.9)	674 532 (54.2)	788 115 (53.6)	827 769 (52.7)	676 423 (50.7)	957 751 (48.0)	541 845 (48.1)	−6.1
10-14	266 804 (21.1)	271 182 (22.1)	354 533 (25.2)	329 659 (26.5)	427 639 (29.1)	500 090 (31.8)	470 026 (35.2)	735 161 (36.9)	424 077 (37.6)	16.5
>14	23 941 (1.9)	24 908 (2.0)	33 251 (2.4)	30 487 (2.4)	41 894 (2.8)	55 341 (3.5)	42 975 (3.2)	72 761 (3.7)	39 168 (3.5)	1.6
No data	27 798 (2.2)	32 629 (2.6)	39 769 (2.8)	31 749 (2.5)	40 276 (2.7)	41 945 (2.7)	44 024 (3.3)	98 084 (4.9)	56 624 (5.0)	2.8
Parity										
Primiparous	820 944 (65.1)	776 433 (63.1)	847 810 (60.2)	689 837 (55.4)	773 104 (52.6)	739 322 (47.1)	644 218 (48.3)	994 114 (49.9)	552 368 (49.0)	−16.1
Multiparous	439 648 (34.8)	453 943 (36.9)	559 821 (39.8)	555 177 (44.6)	698 124 (47.4)	831 864 (52.9)	690 955 (51.7)	998 512 (50.1)	573 988 (51.0)	16.2
No data	1430 (0.1)	163 (0.01)	215 (0.02)	188 (0.02)	212 (0.01)	185 (0.01)	208 (0.02)	964 (0.05)	215 (0.02)	−0.1
Preexisting diseases or prenatal complications										
Prenatal complications	49 512 (3.9)	54 697 (4.4)	63 654 (4.5)	62 292 (5.0)	75 036 (5.1)	90 266 (5.7)	84 071 (6.3)	126 319 (6.3)	80 832 (7.2)	3.3
Preexisting disease	50 295 (4.0)	70 650 (5.8)	101 568 (7.2)	108 552 (8.7)	143 303 (9.7)	184 841 (11.8)	186 573 (14.0)	312 524 (15.7)	197 132 (17.5)	13.5
None of the above	1 162 215 (92.1)	1 105 192 (89.8)	1 242 624 (88.3)	1 074 358 (86.3)	1 253 101 (85.2)	1 296 264 (82.5)	1 064 737 (79.7)	1 554 747 (78.0)	848 607 (75.3)	−16.8
Sex										
Male	663 090 (52.5)	643 797 (52.3)	732 309 (52.0)	650 456 (52.2)	764 070 (51.9)	815 087 (51.9)	691 643 (51.8)	1 030 071 (51.7)	582 025 (51.7)	−0.8
Female	598 932 (47.5)	586 742 (47.7)	675 537 (48.0)	594 746 (47.8)	707 370 (48.1)	756 284 (48.1)	643 738 (48.2)	963 519 (48.3)	544 546 (48.3)	0.8
Births										
Preterm	73 797 (5.8)	72 662 (5.9)	81 503 (5.8)	74 722 (6.0)	89 175 (6.1)	94 251 (6.0)	81 550 (6.1)	120 956 (6.1)	72 539 (6.4)	0.6
Term	1 188 225 (94.2)	1 157 877 (94.1)	1 326 343 (94.2)	1 170 480 (94.0)	1 382 265 (93.9)	1 477 120 (94.0)	1 253 831 (93.9)	1 872 634 (93.9)	1 054 032 (93.6)	−0.6

Together, these variables accounted for more of the observed decrease in the prevalence of mild to moderate SGA birth weight (83.3%) than of the observed decrease in prevalence of severe SGA birth weight (49.7%). The association of all these variables with a decrease in the prevalence of SGA infants between 2012 and 2020 was stronger in the eastern region (relative change, 108.9%) than in the central (relative change, 58.3%) or western region (relative change, 62.4%) (Table 3).

**Table 3.** Relative Associations of Maternal Characteristics With Changes in Prevalence of Infants Born SGA by Region of China

Characteristic	Eastern region		Central region		Western region	
	Absolute change (95% CI)^a^	Relative change, %	Absolute change (95% CI)	Relative change, %	Absolute change (95% CI)	Relative change, %
Hospital level	−0.07 (−0.15 to 0.01)	6.9	0.04 (−0.01 to 0.09)	−2.0	−0.13 (−0.24 to −0.02)	3.8
Maternal education level	−0.22 (−0.38 to −0.05)	21.2	−0.31 (−0.38 to −0.24)	16.1	−0.62 (−0.72 to −0.51)	19.0
Maternal marital status	0.00 (Not applicable)	−0.1	0.00 (Not applicable)	−0.2	0.00 (Not applicable)	−0.3
Maternal age	−0.27 (−0.33 to −0.21)	26.4	−0.40 (−0.44 to −0.35)	20.7	−0.46 (−0.54 to −0.38)	13.8
Prenatal visits	−0.27 (−0.52 to −0.03)	26.8	−0.31 (−0.46 to −0.15)	15.8	−0.57 (−0.81 to −0.33)	17.2
Parity	−0.37 (−0.47 to −0.27)	36.1	−0.36 (−0.41 to −0.30)	18.4	−0.44 (−0.49 to −0.39)	13.2
Preexisting disease or prenatal complications	0.07 (0.00-0.14)	−6.7	0.18 (0.15-0.22)	−9.5	0.13 (0.08-0.18)	−4.1
Sex	0.01 (0.00-0.01)	−0.5	0.01 (0.00-0.01)	−0.5	0.01 (0.00-0.02)	−0.3
Births	0.01 (0.00-0.02)	−1.2	0.01 (0.01-0.02)	−0.7	0.00 (−0.01 to 0.00)	0.1
Total difference	−1.03	100.0	−1.93	100.0	−3.37	100.0
Part explained	−1.12	108.9	−1.12	58.3	−1.90	62.4
Part unexplained	0.09	−8.9	−0.81	41.7	−1.47	37.6

Abbreviation: SGA, small for gestational age.

^a^ Per 100 births.

### Sensitivity Analysis

We obtained similar mean annual rates of decrease and similar profiles of maternal characteristics as in the abovementioned analyses when we defined SGA according to the growth standard from China’s National Health Commission, although using this standard was associated with a higher prevalence of infants born SGA (eTables 2-4 in Supplement 1).

## Discussion

To our knowledge, this is the most detailed analysis so far of the prevalence of infants born SGA in China, permitting stratification based on SGA severity and on regions. Our results indicate that from 2012 to 2020, the overall weighted prevalence of infants born SGA was 6.4% and that the annual prevalence decreased. The decrease was faster for infants with severe SGA birth weight than for those with mild to moderate SGA birth weight. The characteristics most strongly associated with a decrease in the prevalence of infants born SGA were prenatal visits as well as maternal educational level, age, parity, and preexisting disease or prenatal complications. The association of these factors with a decrease in the prevalence of infants born SGA depended on SGA severity and the region under consideration.

The prevalence of infants born SGA across China in 2012 exceeds the 4.6% estimated in a different study^12^ that sampled primarily hospitals in cities where the per capita gross domestic product was at least 10% higher than the national average. The prevalence across the entire study period is comparable to the 6.6% reported for 2020 in North America, Australia, New Zealand, and Europe^2,12,24^ and much lower than the 19.3% reported for 2012 in low- and middle-income countries or the 17.4% reported for 2020 globally.^2,12^ Those previous studies highlight an inverse association between economic development and prevalence of infants born SGA, which we also observed.

We examined the potential association of maternal characteristics with the decrease in the prevalence of infants born SGA, going beyond previous work that focused on temporal trends among mothers stratified by race and ethnicity or among individuals with gestational diabetes.^36,37,38,39^ We found that several maternal factors accounted for two-thirds of the observed decrease in prevalence of infants born SGA between 2012 and 2020. Consistent with our results, previous studies have linked higher risk of infants born SGA with lower maternal educational level,^40^ perhaps because women with more education are more likely to seek and follow medical advice during pregnancy,^41,42,43,44^ and with younger maternal age, which may reflect inadequate maternal physical condition or malnutrition.^45,46^ In contrast to previous work,^47^ this study found a lower risk of infants born SGA among women older than 35 years. A plausible explanation is that women with advanced age, although at intrinsically higher risk of having an infant born SGA, are more likely to comply with medical advice and to maintain a healthy lifestyle, counteracting their higher age-related risk.

Our analysis linked more prenatal visits with lower risk of infants born SGA, consistent with international consensus guidelines.^48^ Such care typically includes maternal and fetal health assessments, health education and guidance, and preventive or therapeutic interventions to mitigate preexisting disease or prenatal complications.^49^ For example, the proportion of prenatal screening institutions in China that measure fetal nuchal translucency in the first trimester increased from 56% in 2015 to 86% in 2020,^50^ which implies increasing use of ultrasonography to determine gestational age. In 2015, the Chinese national guideline began to recommend prophylactic aspirin for pregnant women at high risk of preeclampsia.^51^ These measures are associated with lower risk of infants born SGA.^13,52^ Nevertheless, while a larger number of prenatal visits was associated with lower risk of infants born SGA, there was a limit; risk was higher among women who had more than 15 visits than among those with fewer visits, which may reflect more intensive monitoring due to slow fetal growth, possibly associated with underlying disease.

Preexisting disease or prenatal complications among women in our study were associated with the prevalence of infants born SGA, which highlights the importance of regular prenatal monitoring and appropriate prenatal interventions for such women. Future work should clarify which maternal preexisting diseases or prenatal complications may require more intensive prenatal management to minimize risk of infants born SGA. For example, hypertensive disorders of pregnancy have been shown to increase the risk of infants born SGA,^53^ whereas gestational diabetes may decrease the risk.^54^

The prevalence of severe SGA birth weight decreased faster than that of mild to moderate SGA birth weight. This difference may reflect a focus of screening, diagnosis, and management programs for newborns with severe SGA birth weight.^55,56,57^ Approximately 30% of infants born SGA show abnormal placental function, and most of these births involve severe SGA birth weight, which are targeted by screening and by interventions, such as low-dose aspirin^58^ and antihypertensives,^58^ to treat prenatal complications. Another explanation may be an increase in interventions that shorten gestation, such as elective cesarean delivery, given that the mean gestational age among infants with severe SGA birth weight decreased slightly from 39.0 to 38.4 weeks during the study period. These interventions may shift severe SGA to mild or moderate SGA birth weight, so they may help explain why a greater number of prenatal visits were associated with a lower prevalence of severe SGA birth weight than a lower prevalence of mild to moderate SGA birth weight in our sample.

Purely sociodemographic characteristics of women (educational level, age, and parity) have been associated less with the prevalence of severe SGA birth weight than with the prevalence of mild to moderate SGA birth weight (50% vs 83%), which may reflect specific biochemical or cellular factors that cause severe SGA birth weight by compromising placental function. The same factors may also explain why women in our sample with prenatal complications experienced a higher rate of severe SGA than mild to moderate SGA birth weight. Future work should explore whether increasing the number of prenatal visits can improve early detection, management, and even prevention of prenatal complications.

There was a faster decrease in the prevalence of infants born SGA in less-developed western China than in more developed eastern regions in this study. Our observation that maternal characteristics have been less associated with the decrease in the prevalence of infants born SGA in less developed regions may be explained by the improvement in diet and nutrition intake of pregnant women in these regions.^59,60^ Other possible explanations are improvements in health conditions and urbanization, which imply increases in health awareness, access to health care, and quality of prenatal care. Future research should explore what factors have been associated with the decreasing prevalence of infants born SGA in central and western China, which may help policymakers tailor interventions regionally.

### Limitations

Our findings should be interpreted with caution given several limitations. One is that our sample was biased toward urban women, among whom the risk of delivering an infant classified as SGA should be lower because of better nutrition and access to comprehensive prenatal care. We tried to minimize urban bias by weighting prevalence according to the distribution of live births between urban and rural settings. Another limitation is that gestational age among a substantial proportion of infants was estimated based on the last menstrual period rather than on more accurate prenatal ultrasonography.^52,61,62^ Nevertheless, the gestational ages determined by the 2 techniques tended to agree within 1 week.^63^ A third limitation is that we were unable to take into account several variables that were missing from the NMNMSS, such as fetal complications or maternal nutritional status and body mass index.

## Conclusions

In this cross-sectional study of singleton live births in China, the prevalence of infants born SGA decreased from 2012 through 2020; this decrease was associated with changes in maternal characteristics. The insights from this work may help target public health campaigns and interventions to specific subsets of women in particular parts of the country to further reduce the risk of infants born SGA.

## Notes

Supplement 1. **eAppendix 1.** Sampling Methods
eReferences.
**eAppendix 2.** Weighting Methods **eFigure.** Flowchart of Data Inclusion **eTable 1.** Distribution of Live Births in China and NMNMSS Between Urban and Rural Settings in 2020 **eTable 2.** Prevalence of Small for Gestational Age in China From 2012 Through 2020, Based on the Local National Growth Standards **eTable 3.** Relative Associations of Maternal Characteristics With Changes in Prevalence of Small for Gestational Age Stratified by Severity, Based on the Local National Growth Standards **eTable 4.** Relative Associations of Maternal Characteristics With Changes in Prevalence of Small for Gestational Age Stratified by Region, Based on the Local National Growth Standards

Supplement 2.
Data Sharing Statement
