# Supplementary material for: Maternal Characteristics and Prevalence of Infants Born Small for Gestational Age
Source: JAMA Netw Open. 2024 Aug 21;7(8):e2429434. doi: 10.1001/jamanetworkopen.2024.29434 (PMC11339661; doi:10.1001/jamanetworkopen.2024.29434)
Supplement: Supplement 1. — eAppendix 1. Sampling Methods eReferences. eAppendix 2. Weighting Methods eFigure. Flowchart of Data Inclusion eTable 1. Distribution of Live Births in China and NMNMSS Between Urban and Rural Settings in 2020 eTable 2. Prevalence of Small for Gestational Age in China From 2012 Through 2020, Based on the Local National Growth Standards eTable 3. Relative Associations of Maternal Characteristics With Changes in Prevalence of Small for Gestational Age Stratified by Severity, Based on the Local National Growth Standards eTable 4. Relative Associations of Maternal Characteristics With Changes in Prevalence of Small for Gestational Age Stratified by Region, Based on the Local National Growth Standards [file jamanetwopen-e2429434-s001.pdf]

# Supplementary Online Content

Xiang L, Li X, Mu Y, et al. Maternal characteristics and prevalence of infants born small for gestational age. *JAMA Netw Open.* 2024;7(8):e2429434. doi:10.1001/jamanetworkopen.2024.29434

**eAppendix 1.** Sampling Methods
**eReferences.**
**eAppendix 2.** Weighting Methods
**eFigure.** Flowchart of Data Inclusion
**eTable 1.** Distribution of Live Births in China and NMNMSS Between Urban and Rural Settings in 2020
**eTable 2.** Prevalence of Small for Gestational Age in China From 2012 Through 2020, Based on the Local National Growth Standards
**eTable 3.** Relative Associations of Maternal Characteristics With Changes in Prevalence of Small for Gestational Age Stratified by Severity, Based on the Local National Growth Standards
**eTable 4.** Relative Associations of Maternal Characteristics With Changes in Prevalence of Small for Gestational Age Stratified by Region, Based on the Local National Growth Standards

This supplementary material has been provided by the authors to give readers additional information about their work.

© 2024 Xiang L et al. *JAMA Network Open.*

## **eAppendix 1.** Sampling Methods

The National Maternal Near Miss Surveillance System (NMNMSS) in China, a facility-based surveillance system, was established in October 2010. The sample framework of the NMNMSS has been detailed earlier.[1-4] Urban districts and rural counties were determined based on the National Maternal and Child Mortality Surveillance System (NMCMSS) in China. The NMCMSS was a population-based registration system for maternal and child deaths established by the Ministry of Health of China in 1996. The NMCMSS utilized a stratified random sampling method based on 17 socio-economic strata in China, including 176 urban districts and rural counties. [5] Over the years, with the decline in maternal and child mortality rates in China, the NMCEMM added 30 urban districts and 130 rural counties to increase the national representation. Therefore, the NMCMSS covered 127 urban districts and 209 rural counties. The NMCMSS can provide key maternal and child death metrics as nationally reprehensive maternal and child health indicators, such as maternal mortality, neonatal mortality, and under-5 child mortality. In 2010, the NMNMSS included 326 urban districts and rural counties, with 273 selected based on regional strata and urban or rural characteristics from the NMCMSS. Additionally, 53 urban districts and rural counties belonging to provincial Maternal and Child Mortality Surveillance System were randomly sampled across all strata to ensure representation of urban and rural populations in all three regions of China (eastern, central, and western). The surveillance sites of the NMNMSS has been published in previous study.[3] Finally, based on the World Health Organization's criteria for selecting health facilities for monitoring maternal and perinatal health globally,[5] once surveillance sites were selected, two health facilities with more than 1000 deliveries per year were randomly selected in these areas (if only one facility was avaialable, then that facility was selected).

**eReferences.**

1. Mu Y, Wang X, Li X, et al. The national maternal near miss surveillance in China: A facility-based surveillance system covered 30 provinces. Medicine 2019; 98(44): e17679.
2. Zhu J, Liang J, Mu Y, et al. Sociodemographic and obstetric characteristics of stillbirths in China: a census of nearly 4 million health facility births between 2012 and 2014. The Lancet Global health 2016; 4(2): e109-18.
3. Liang J, Mu Y, Li X, et al. Relaxation of the one child policy and trends in caesarean section rates and birth outcomes in China between 2012 and 2016: observational study of nearly seven million health facility births. Bmj 2018; 360: k817.
4. Deng K, Liang J, Mu Y, et al. Preterm births in China between 2012 and 2018: an observational study of more than 9 million women. Lancet Glob Health. 2021 Sep;9(9):e1226-e1241.
5. Feng XL, Guo S, Hipgrave D, et al. China's facility-based birth strategy and neonatal mortality: a population-based epidemiological study. Lancet 2011; 378(9801): 1493-500.
6. Shah A, Faundes A, Machoki M, et al. Methodological considerations in implementing the WHO Global Survey for Monitoring Maternal and Perinatal Health. Bulletin of the World Health Organization 2008; 86(2): 126-31.

© 2024 Xiang L et al. *JAMA Network Open.*

## **eAppendix 2.** Weighting Methods

Firstly, based on the data from the China's 6th National Census in 2010 and the China's 7th National Census in 2020, we obtained the urban/rural ratio of newborns in different provinces in 2010 and 2020, respectively. Secondly, we calculated the average speed of increase in urban/rural ratio for each province over the past 11 years and then estimated the urban/rural ratio for each province from 2011 to 2019. Thirdly, we obtained the number of urban and rural births based on the birth population and urban/rural ration in various provinces from 2012 to 2019. Finally, we weighted the data in this study by the number of urban and rural births in each province annually. **eTable 1** showed the the births in various provinces in both China and NMNMSS in 2020 and the corresponding weights.

© 2024 Xiang L et al. *JAMA Network Open.*

**eFigure.** Flowchart of Data Inclusion

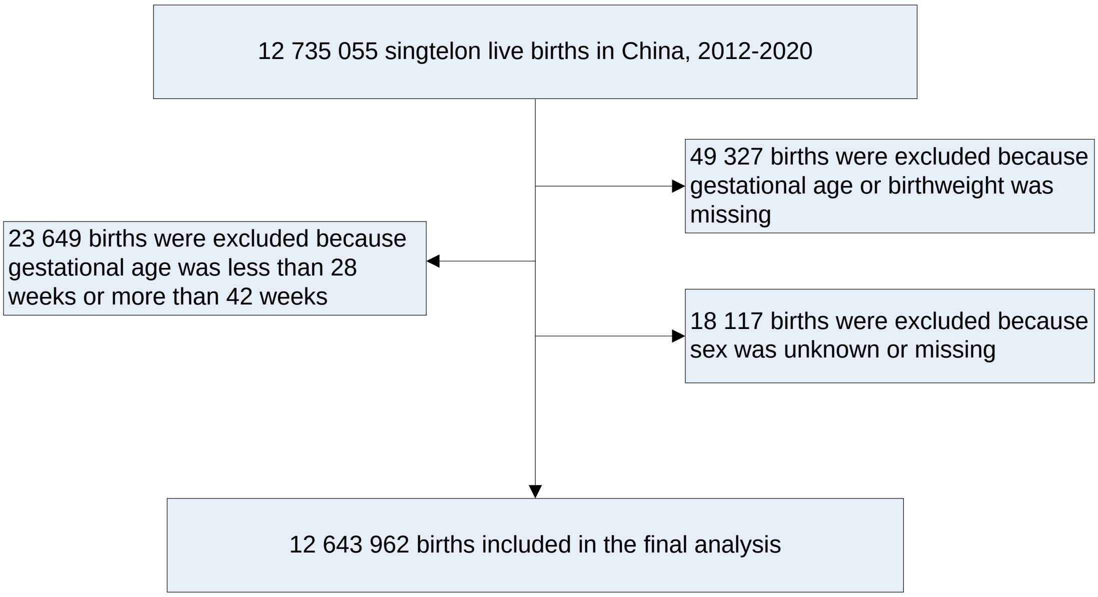


© 2024 Xiang L et al. *JAMA Network Open.*

**eTable 1.** Distribution of Live Births in China and NMNMSS Between Urban and Rural Settings in 2020

| Province | Stratification | Births (%) in China in 2020 | Births (%) in NMNMSS in 2020 | Weighted |
|---|---|---|---|---|
| Beijing | Urban | 152730 (1.3) | 7514 (0.7) | 1.92 |
| | Rural | - | - | - |
| Tianjin | Urban | 83049 (0.7) | 20352 (1.8) | 0.39 |
| | Rural | - | - | - |
| Hebei | Urban | 186014 (1.6) | 17235 (1.5) | 1.02 |
| | Rural | 421420 (3.5) | 16607 (1.5) | 2.39 |
| Shanxi | Urban | 115534 (1.0) | 27189 (2.4) | 0.40 |
| | Rural | 172213 (1.4) | 12389 (1.1) | 1.31 |
| Inner Mongolia | Urban | 79061 (0.7) | 11542 (1.0) | 0.65 |
| | Rural | 93890 (0.8) | 2228 (0.2) | 3.98 |
| Liaoning | Urban | 138344 (1.2) | 26126 (2.3) | 0.50 |
| | Rural | 81736 (0.7) | 3563 (0.3) | 2.17 |
| Jilin | Urban | 53673 (0.4) | 31932 (2.8) | 0.16 |
| | Rural | 62766 (0.5) | 4067 (0.4) | 1.46 |
| Heilongjiang | Urban | 60156 (0.5) | 13292 (1.2) | 0.43 |
| | Rural | 59537 (0.5) | 3235 (0.3) | 1.74 |
| Shanghai | Urban | 122565 (1.0) | 40169 (3.6) | 0.29 |
| | Rural | - | - | - |
| Jiangsu | Urban | 286860 (2.4) | 16301 (1.4) | 1.66 |
| | Rural | 276352 (2.3) | 18426 (1.6) | 1.42 |
| Zhejiang | Urban | 217260 (1.8) | 34978 (3.1) | 0.59 |
| | Rural | 241849 (2.0) | 28154 (2.5) | 0.81 |
| Anhui | Urban | 188905 (1.6) | 29040 (2.6) | 0.61 |
| | Rural | 385977 (3.2) | 24943 (2.2) | 1.46 |
| Fujian | Urban | 153319 (1.3) | 29670 (2.6) | 0.49 |
| | Rural | 227674 (1.9) | 10016 (0.9) | 2.15 |
| Jiangxi | Urban | 141721 (1.2) | 24025 (2.1) | 0.56 |
| | Rural | 285101 (2.4) | 18930 (1.7) | 1.42 |
| Shandong | Urban | 358163 (3.0) | 21135 (1.9) | 1.60 |
| | Rural | 509646 (4.3) | 8898 (0.8) | 5.41 |
| Henan | Urban | 227177 (1.9) | 52081 (4.6) | 0.41 |
| | Rural | 688268 (5.8) | 51226 (4.5) | 1.27 |
| Hubei | Urban | 228521 (1.9) | 15683 (1.4) | 1.38 |
| | Rural | 248259 (2.1) | 11557 (1.0) | 2.03 |
| Hunan | Urban | 146162 (1.2) | 33988 (3.0) | 0.41 |
| | Rural | 419355 (3.5) | 29211 (2.6) | 1.35 |
| Guangdong | Urban | 839921 (7.0) | 12225 (1.1) | 6.48 |
| | Rural | 450827 (3.8) | 31995 (2.8) | 1.33 |
| Guangxi | Urban | 227325 (1.9) | 28408 (2.5) | 0.76 |

© 2024 Xiang L et al. *JAMA Network Open.*

| Province | Stratification | Births (%) in China in 2020 | Births (%) in NMNMSS in 2020 | Weighted |
|---|---|---|---|---|
| | Rural | 340053 (2.8) | 33226 (2.9) | 0.97 |
| Hainan | Urban | 72887 (0.6) | 31868 (2.8) | 0.22 |
| | Rural | 31143 (0.3) | 7066 (0.6) | 0.42 |
| Chongqing | Urban | 152258 (1.3) | 34335 (3.0) | 0.42 |
| | Rural | 87168 (0.7) | 3516 (0.3) | 2.34 |
| Sichuan | Urban | 283296 (2.4) | 11457 (1.0) | 2.33 |
| | Rural | 351266 (2.9) | 6442 (0.6) | 5.15 |
| Guizhou | Urban | 178745 (1.5) | 29956 (2.7) | 0.56 |
| | Rural | 346635 (2.9) | 12861 (1.1) | 2.54 |
| Yunnan | Urban | 171608 (1.4) | 27295 (2.4) | 0.59 |
| | Rural | 343570 (2.9) | 13703 (1.2) | 2.37 |
| Shaanxi | Urban | 182955 (1.5) | 49373 (4.4) | 0.35 |
| | Rural | 170038 (1.4) | 4521 (0.4) | 3.55 |
| Gansu | Urban | 93789 (0.8) | 26856 (2.4) | 0.33 |
| | Rural | 169222 (1.4) | 9172 (0.8) | 1.74 |
| Qinghai | Urban | 21837 (0.2) | 10549 (0.9) | 0.20 |
| | Rural | 45417 (0.4) | 2484 (0.2) | 1.73 |
| Ningxia | Urban | 42924 (0.4) | 11609 (1.0) | 0.35 |
| | Rural | 40103 (0.3) | 8801 (0.8) | 0.43 |
| Xinjiang | Urban | 90695 (0.8) | 16176 (1.4) | 0.53 |
| | Rural | 88029 (0.7) | 6975 (0.6) | 1.19 |
| | Total | 11937069 (100.0) | 1126571 (100.0) | - |

© 2024 Xiang L et al. *JAMA Network Open.*

**eTable 2.** Prevalence of Small for Gestational Age in China From 2012 Through 2020, Based on the Local National Growth Standards

| Geographic area and SGA severity | SGA infants, No. (weighted % of total [a]) | | | | | | | | | | Annual decrease rate, mean (95% CI), % [b] |
|---|---|---|---|---|---|---|---|---|---|---|---|
| | Entire period | 2012 | 2013 | 2014 | 2015 | 2016 | 2017 | 2018 | 2019 | 2020 | |
| **Entire country** | | | | | | | | | | | |
| All severities | 1 043 513 (8.4) | 117 481 (9.6) | 115 970 (9.7) | 126 729 (9.2) | 112 578 (9.2) | 117 392 (8.1) | 124 931 (8.0) | 102 245 (7.9) | 147 794 (7.6) | 78 393 (7.1) | 3.9 (3.1-4.3) |
| Severe | 300 775 (2.4) | 36 177 (3.0) | 35 281 (2.9) | 37 763 (2.7) | 33 147 (2.7) | 33 760 (2.3) | 35 029 (2.2) | 28 205 (2.2) | 40 797 (2.1) | 20 616 (1.8) | 5.5 (4.5-6.4) |
| Mild to moderate | 742 738 (6.0) | 81 304 (6.6) | 80 689 (6.8) | 88 966 (6.5) | 79 431 (6.5) | 83 632 (5.8) | 89 902 (5.8) | 74 040 (5.7) | 106 997 (5.5) | 57 777 (5.3) | 3.0 (2.5-3.5) |
| **Eastern region** | | | | | | | | | | | |
| All severities | 246 230 (7.3) | 27 072 (7.7) | 26 229 (8.0) | 30 675 (7.7) | 26 832 (7.8) | 27 088 (7.1) | 29 626 (7.1) | 24 841 (7.1) | 35 308 (7.0) | 18 559 (6.5) | 2.1 (1.0-3.2) |
| Severe | 64 627 (1.9) | 7 609 (2.1) | 7 279 (2.2) | 8 392 (2.1) | 7 153 (2.1) | 7 213 (2.0) | 7 479 (1.8) | 6 222 (1.8) | 8 824 (1.8) | 4 456 (1.5) | 3.7 (2.3-5.1) |
| Mild to moderate | 181 603 (5.4) | 19 463 (5.6) | 18 950 (5.8) | 22 283 (5.6) | 19 679 (5.7) | 19 875 (5.1) | 22 147 (5.3) | 18 619 (5.3) | 26 484 (5.2) | 14 103 (5.0) | 1.5 (0.4-2.6) |
| **Central region** | | | | | | | | | | | |
| All severities | 382 333 (7.7) | 42 697 (8.8) | 42 118 (8.7) | 46 350 (8.5) | 39 800 (8.3) | 42 078 (7.4) | 44 597 (7.3) | 38 907 (7.3) | 56 159 (7.0) | 29 627 (6.3) | 3.9 (3.0-4.7) |
| Severe | 109 755 (2.2) | 13 130 (2.7) | 12 581 (2.6) | 13 598 (2.5) | 11 591 (2.4) | 11 940 (2.1) | 12 332 (2.0) | 10 811 (2.0) | 15 859 (2.0) | 7 913 (1.7) | 5.1 (2.9-7.3) |
| Mild to moderate | 272 578 (5.5) | 29 567 (6.1) | 29 537 (6.1) | 32 752 (6.0) | 28 209 (5.9) | 30 138 (5.3) | 32 265 (5.3) | 28 096 (5.3) | 40 300 (5.0) | 21 714 (4.6) | 3.4 (2.8-3.9) |
| **Western region** | | | | | | | | | | | |

© 2024 Xiang L et al. *JAMA Network Open.*

| Geographic area and SGA severity | SGA infants, No. (weighted % of total [a]) | | | | | | | | | | Annual decrease rate, mean (95% CI), % [b] |
|---|---|---|---|---|---|---|---|---|---|---|---|
| | Entire period | 2012 | 2013 | 2014 | 2015 | 2016 | 2017 | 2018 | 2019 | 2020 | |
| All severities | 414 950 (10.8) | 47 712 (13.0) | 47 623 (12.8) | 49 704 (12.0) | 45 946 (11.8) | 48 226 (10.5) | 50 708 (10.2) | 38 497 (9.7) | 56 327 (9.3) | 30 207 (8.8) | 5.0 (4.2-5.8) |
| Severe | 126 393 (3.3) | 15 438 (4.3) | 15 421 (4.2) | 15 773 (3.9) | 14 403 (3.8) | 14 607 (3.2) | 15 218 (3.1) | 11 172 (2.9) | 16 114 (2.7) | 8 247 (2.4) | 7.0 (5.9-8.0) |
| Mild-moderate | 288 557 (7.5) | 32 274 (8.7) | 32 202 (8.6) | 33 931 (8.1) | 31 543 (8.0) | 33 619 (7.3) | 35 490 (7.1) | 27 325 (6.8) | 40 213 (6.6) | 21 960 (6.4) | 4.1 (3.3-4.8) |

Abbreviation: SGA, small for gestational age.

[a] Adjusted for the distribution of live births between urban and rural settings.

[b] Mean annual decrease rate over the entire period, as estimated by log-linear Poisson regression involving robust variance and adjustment for the distribution of live births between urban and rural settings and clustering of births within hospitals.

© 2024 Xiang L et al. *JAMA Network Open.*

**eTable 3.** Relative Associations of Maternal Characteristics With Changes in Prevalence of Small for Gestational Age Stratified by Severity, Based on the Local National Growth Standards

| Characteristics | SGA | | Severe SGA | | Mild to moderate SGA | |
|---|---|---|---|---|---|---|
| | **Absolute change (95% CI)[a]** | **Relative change, %** | **Absolute change (95% CI)** | **Relative change, %** | **Absolute change (95% CI)** | **Relative change, %** |
| Regions | 0.05 (0.01-0.09) | -2.2 | 0.02 (0.00-0.03) | -1.4 | 0.04 (0.02-0.07) | -2.8 |
| Hospital level | -0.07 (-0.14 to 0.01) | 2.7 | -0.01 (-0.04 to 0.02) | 0.9 | -0.06 (-0.11 to -0.01) | 3.8 |
| Maternal educational level | -0.48 (-0.58 to -0.37) | 19.2 | -0.18 (-0.22 to -0.14) | 16.1 | -0.30 (-0.37 to -0.22) | 19.8 |
| Maternal marital status | 0.00 (0.00-0.01) | -0.2 | 0.00 (not applicable) | -0.1 | 0.00 (not applicable) | -0.2 |
| Maternal age | -0.50 (-0.54 to -0.45) | 20.0 | -0.15 (-0.16 to -0.13) | 13.1 | -0.35 (-0.38 to -0.32) | 25.6 |
| Prenatal visits | -0.46 (-0.64 to -0.28) | 18.4 | -0.22 (-0.31 to -0.14) | 20.1 | -0.23 (-0.35 to -0.10) | 16.5 |
| Parity | -0.50 (-0.55 to -0.44) | 20.0 | -0.16 (-0.18 to -0.13) | 13.9 | -0.34 (-0.37 to -0.30) | 24.6 |
| Preexisting disease or prenatal complications | 0.09 (0.05-0.13) | -3.7 | 0.09 (0.07-0.11) | -7.7 | 0.01 (-0.02 to 0.04) | -0.4 |
| Sex | 0.00 (not applicable) | 0.2 | 0.00 (not applicable) | 0.04 | 0.00 (-0.01 to 0.00) | 0.9 |
| Births | -0.02 (-0.02 to -0.01) | 0.6 | 0.00 (not applicable) | 0.4 | -0.01 (-0.02 to 0.00) | 0.9 |
| Total difference | -2.48 | 100.0 | -1.12 | 100.0 | -1.49 | 100.0 |
| Part explained | -1.86 | 75.1 | -0.62 | 55.2 | -1.24 | 89.6 |
| Part unexplained | -0.62 | 24.9 | -0.50 | 44.8 | -0.26 | 10.4 |

Abbreviation, SGA, small for gestational age.

[a] Per 100 births.

© 2024 Xiang L et al. *JAMA Network Open.*

**eTable 4.** Relative Associations of Maternal Characteristics With Changes in Prevalence of Small for Gestational Age Stratified by Region, Based on the Local National Growth Standards

| Characteristic | Eastern region | | Central region | | Western region | |
|---|---|---|---|---|---|---|
| | Absolute change (95% CI)[a] | Relative change, % | Absolute change (95% CI) | Relative change, % | Absolute change (95% CI) | Relative change, % |
| Hospital level | -0.08 (-0.18 to 0.02) | 5.1 | 0.03 (-0.03 to 0.09) | -1.3 | -0.17 (-0.34 to 0.00) | 4.1 |
| Maternal educational level | -0.28 (-0.49 to -0.08) | 18.2 | -0.39 (-0.49 to -0.30) | 15.6 | -0.75 (-0.89 to -0.61) | 18.1 |
| Maternal marital status | 0.00 (not applicable) | -0.1 | 0.00 (0.00-0.01) | -0.1 | 0.01 (0.00-0.01) | -0.2 |
| Maternal age | -0.38 (-0.46 to -0.31) | 31.0 | -0.53 (-0.58 to -0.47) | 21.2 | -0.58 (-0.68 to -0.49) | 14.1 |
| Prenatal visits | -0.27 (-0.57 to -0.04) | 21.8 | -0.36 (-0.54 to -0.17) | 14.2 | -0.67 (-0.99 to -0.36) | 16.3 |
| Parity | -0.49 (-0.62 to -0.36) | 39.6 | -0.47 (-0.54 to -0.40) | 18.8 | -0.54 (-0.60 to -0.48) | 13.0 |
| Preexisting disease or prenatal complications | 0.02 (-0.06 to 0.11) | -2.0 | 0.14 (0.10-0.18) | -5.7 | 0.08 (0.02-0.14) | -2.0 |
| Sex | 0.00 (not applicable) | 0.2 | -0.01 (-0.01 to 0.00) | 0.3 | 0.00 (-0.01 to 0.00) | 0.1 |
| Births | -0.01 (-0.02 to 0.00) | 0.7 | -0.01 (-0.01 to 0.00) | 0.3 | -0.03 (-0.05 to -0.02) | 0.8 |
| Total difference | -1.23 | 100.0 | -2.50 | 100.0 | -4.14 | 100.0 |
| Part explained | -1.48 | 120.5 | -1.58 | 63.4 | -2.66 | 64.3 |
| Part unexplained | 0.25 | -20.5 | -0.92 | 36.6 | -1.48 | 35.7 |

Abbreviation, SGA, small for gestational age.

[a] Per 100 births.

© 2024 Xiang L et al. *JAMA Network Open.*
