# Supplementary material for: Maternal Characteristics and Prevalence of Infants Born Small for Gestational Age
Source: JAMA Netw Open. 2024 Aug 21;7(8):e2429434. doi: 10.1001/jamanetworkopen.2024.29434 (PMC11339661; doi:10.1001/jamanetworkopen.2024.29434)
Supplement: Supplement 2. — Data Sharing Statement [file jamanetwopen-e2429434-s002.pdf]

Data Sharing Statement

Xiang. Maternal Characteristics and Prevalence of Infants Born Small for Gestational Age. *JAMA Netw Open*. Published August 21, 2024. doi:10.1001/jamanetworkopen.2024.29434

Data
Data available: No
